# Depression, Stress, and Suicide in Korean Adults before and during the COVID-19 Pandemic Using Data from the Korea National Health and Nutritional Examination Survey

**DOI:** 10.3390/jpm12081305

**Published:** 2022-08-11

**Authors:** So Young Kim, Dae Myoung Yoo, Mi Jung Kwon, Ji Hee Kim, Joo-Hee Kim, Jee Hye Wee, Hyo Geun Choi

**Affiliations:** 1 Bundang CHA Medical Center, Department of Otorhinolaryngology-Head and Neck Surgery, CHA University, Seongnam 13488, Korea; 2 Hallym Data Science Laboratory, Hallym University College of Medicine, Anyang 14066, Korea; 3 Department of Pathology, Hallym Sacred Heart Hospital, Hallym University College of Medicine, Anyang 14068, Korea; 4 Department of Neurosurgery, Hallym University College of Medicine, Anyang 14068, Korea; 5 Department of Medicine, Division of Pulmonary, Allergy, and Critical Care Medicine, Hallym Sacred Heart Hospital, Hallym University College of Medicine, Anyang 14068, Korea; 6 Department of Otorhinolaryngology-Head and Neck Surgery, Hallym University College of Medicine, Anyang 14068, Korea

**Keywords:** depression, COVID-19, risk factors, cohort studies, epidemiology

## Abstract

This study investigated changes in the prevalence of depression, stress, and suicidal attempts during the COVID-19 pandemic. The ≥19-year-old population in the Korea National Health and Nutrition Examination Survey in 2019 and 2020 was included. The histories of depression, stress, and suicidal attempts were compared between the 2019 and 2020 cohorts using multiple logistic regression analysis with complex sampling. The prevalence of depression was not significantly different between the 2019 and 2020 groups (4.1% vs. 4.5%, *p* = 0.326). The prevalence of stress and suicide attempts was also not significantly different between groups (all *p* > 0.05). The rates of depression, stress, and suicide attempts were not associated with the 2020 group compared to the 2019 group (all *p* > 0.05). The 19- to 39-year-old group in the 2020 group indicated a higher rate of depression (diagnosed by physicians) than the 19- to 39-year-old group in the 2019 group (adjusted odds ratio = 1.58, 95% confidence intervals = 1.00–2.50, *p* = 0.049). The risks of depression, stress, and suicidal attempts were not related to the COVID-19 pandemic in Korean adults. A young adult population demonstrated an increased risk of depression associated with the COVID-19 pandemic.

## 1. Introduction

The COVID-19 pandemic has been reported to impact mental health [1]. Direct viral effects on the central nervous system have been reported through the increased risk of neural inflammation and neurologic impacts in postmortem cases [2,3]. In addition, mental distress during the COVID-19 pandemic increased compared to the trends of previous years in the UK population [4]. In particular, young and female subpopulations indicated a notable elevation of mental distress during the COVID-19 pandemic [4]. They suggested that pre-existing inequalities in socioeconomic factors, such as age, sex, employment, and house type, may impose the risk of mental distress [4]. Thus, these factors should be included when investigating the association of the COVID-19 pandemic with mental health. In addition, many online surveys based on nonprobability sampling harbor a risk of bias; therefore, random sampling and selection of representative cohorts are important for evaluating mental health research during the COVID-19 pandemic [5].

The affective disorders of depression, stress, and suicide have been the focus of many risk assessment studies during the COVID-19 pandemic [6,7,8]. Due to the uncertainty and fear of novel viral diseases, an increased risk of depression during the COVID-19 pandemic can be predicted [7]. In the older adult population of Korea, the fear of COVID-19 and poor understanding of public health measures were related to higher odds for anxiety and depression [9]. In addition, adolescents also demonstrated higher rates of depressive and anxiety symptoms associated with fear of COVID-19 infection [10]. The changes in lifestyle due to quarantine measures were suggested as one of the factors contributing to the increased risk of depression [7]. The impact of the COVID-19 pandemic on the risk of depression and suicide can be different according to age and sex due to differences in mindset and access to information [11,12]. According to mindsponge theory, individuals with specific mindsets and access to different sorts of information can absorb information, such as stresses caused by the COVID-19 pandemic, differently [11,12].

Korea was reported to have the greatest suicide rate among nations of the Organization of Economic Cooperation and Development [13]. In addition, the prevalence of depression in Korea increased to 5.3% in 2013 and 2.8% in 2002 [14]. The increased risk of depression related to the COVID-19 pandemic could impair quality of life and health, as well as elevate socioeconomic burdens. Thus, it may be important to estimate the changes in depression, stress, and suicide during the COVID-19 pandemic compared to the pre-pandemic era. Although a few recent studies reported increased depression during the COVID-19 pandemic, many studies were based on online surveys and did not consider confounders of socioeconomic and lifestyle factors.

This study hypothesized that there may be differences in the association of the COVID-19 pandemic with depression, stress, and suicide attempts according to age and sex. Because socioeconomic factors can influence the risks of depression, stress, and suicidal attempts, these factors were adjusted in the analyses to evaluate the relation of the COVID-19 pandemic with depression, stress, and suicide attempts. To test this, we compared populations before and during the COVID-19 pandemic for rates of depression, stress, and suicide attempts with adjustments for socioeconomic factors in addition to lifestyle and comorbidities.

## 2. Materials and Methods

### 2.1. Study Population and Data Collection

This study was exempt from institutional ethical review from the Institutional Review Board based on the bioethics law of Korea, which indicated exemption from ethical review for the study conducted by the Korean national government, in order to help improve public health. All Korea National Health and Nutrition Examination Survey (KNHNES) data analyses were conducted in accordance with the guidelines and regulations provided by the Institutional Review Board of the Centers for Disease Control and Prevention of Korea (KCDC). The understanding, reliability, and validity of each question were investigated by the KCDC to verify the applicability of the surveys [15].

This cross-sectional study used data from the KNHNES and covered the nation using statistical methods based on designed sampling and adjusted weighted values. The data were collected by the Centers for Disease Control and Prevention of Korea. Each year, a panel selected 192 enumeration districts and 25 households in each district for proper sampling to reflect the entire Korean population. Sampling was weighted by statisticians, who performed poststratification analyses and considered nonresponse rates and extreme values. The eighth KNHANES from 2019 (1 January 2019–31 December 2019) and 2020 (1 January 2020–31 December 2020) was analyzed. Details of the sampling methods are described on the KNHNES website [15].

**Figure 1:**
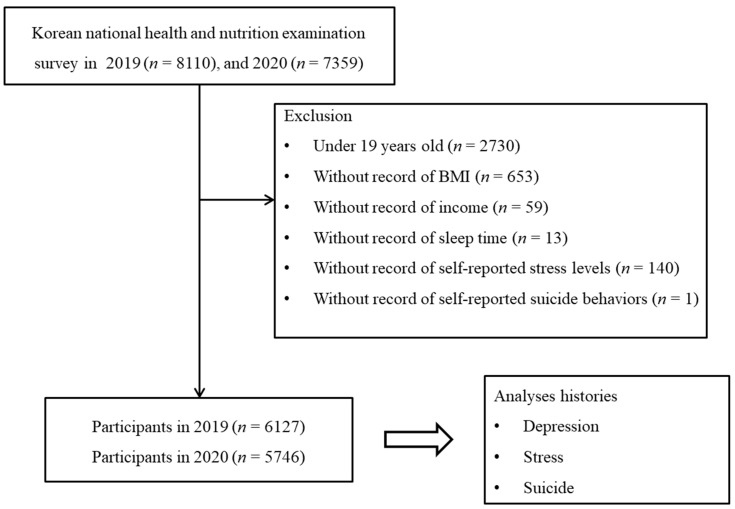
Study design of the present study. A total of 6127 participants from the 2019 cohort population and 5746 from the 2020 cohort population were compared for histories of depression, stress, and suicidal attempts.

Of the 15,469 total participants (8110 in 2019; 7359 in 2020), the following were excluded from this study for the indicated reasons: participants under 19 years old who did not complete the questionnaire for allergic diseases (*n* = 2730), those without records of body mass index (BMI, *n* = 653), those without recorded income (*n* = 59), those who did report sleep time (*n* = 13), those without self-reported stress levels (*n* = 140), and those lacking information regarding self-reported suicide behaviors (*n* = 1). Finally, 11,873 participants (6217 in 2019; 5746 in 2020) 19 through 80+ years old were included in this study (Figure 1). We then analyzed the self-reported prevalence of depression, stress, and suicide behaviors between 2019 and 2020.

### 2.2. Survey

Exposure

In 2019 and 2020, adult participants were selected, as stated above, to represent the entire population in Korea. The participants in 2019 had no follow-up. The 2020 participants were newly selected from the entire Korean population.

Outcome

Participants were asked about their histories of depression, stress levels, and suicidal behaviors. Regarding depression, participants were asked the following two questions: “Have you ever been diagnosed with depression by a doctor?” and “Are you currently treated with depression?”. If yes, it was defined as ‘doctor-diagnosed depression’ and ‘current depression’, respectively. Regarding stress levels, participants were asked the following question: “How much do you feel the stress in daily living?”. The answers are as follows: very severe, a lot, a little, none. Regarding suicidal behaviors, participants were asked the following two questions: “Have you ever planned suicide specifically within 1 year?” and “Have you ever attempted suicide within 1 year?”. If yes, it was defined as ‘planning suicide’ and ‘attempting suicide’, respectively.

Covariate

Income was recalculated by dividing total household income by the square root of the number of household members [16]. Employment status was classified as unemployed or employed. Educational status was divided as elementary school or under, unknown; junior high school; high school; and college or over. The type of house was surveyed as detached house, condominium, townhouse, and others. Marriage status was divided into married, unmarried, and unknown. BMI (kg/m^2^) was calculated using height and weight. Smoking status (nonsmoker, past smoker, current smoker) and alcohol consumption (nonconsumer, 1 to 5 times/month, ≥2 times/week) were surveyed. Sleep time was calculated as 5/7 times on weekdays plus 2/7 times on weekends [16]. Data regarding hypertension, dyslipidemia, stroke, ischemic heart disease, diabetes mellitus, and chronic kidney disease were collected by asking whether participants had received a doctor’s diagnosis of each disease.

### 2.3. Statistical Analysis

The general characteristics in 2019 and 2020 were compared using linear regression analysis with complex sampling and the chi-square test with Rao–Scott correction to represent the entire population, as this study was designed to use weighted values [15].

The odds ratios (ORs) for depression, stress levels, and suicide behaviors in 2020 compared to 2019 were calculated using multiple logistic regression analysis with complex sampling. Crude and adjusted (continuous: age, income, BMI, and sleep time; categorical: sex, employment, education, house type, marital status, smoking, alcohol consumption, hypertension, dyslipidemia, stroke, ischemic heart disease, diabetes mellitus, and chronic kidney disease) models were designed. Subgroup analyses for depression by age and sex were analyzed.

Two-tailed analyses were conducted and *p* values lower than 0.05 were considered to indicate significance; 95% confidence intervals (CIs) were also calculated. The weights recommended by the KNHNES were applied, and all results are thus presented as weighted values. The data were analyzed using SPSS ver. 25.0 (IBM, Armonk, NY, USA).

## 3. Results

The history of depression was not different between the 2019 and 2020 groups (4.1% vs. 4.5%, *p* = 0.326, Table 1). The levels of stress and suicide attempts were also comparable between the 2019 and 2020 groups (all *p* > 0.05). The average age was 51.7 years old (standard deviation [SD] = 16.9) for the 2019 group and 52.0 years old (SD = 17.3) for the 2020 group (*p* = 0.799). Age groups, sex, income, income groups, employment, educational status, house type, marriage status, alcohol consumption, sleep duration, and histories of hypertension, stroke, ischemic heart disease, and diabetes mellitus were not different between the 2019 and 2020 groups (all *p* > 0.05). Body mass index was higher in the 2020 group than in the 2019 group (24.2 [SD = 3.7] vs. 23.9 [SD = 3.6], *p* = 0.001). The rates of dyslipidemia and chronic kidney disease were higher in the 2020 group than in the 2019 group (all *p* < 0.05).

The odds of depression diagnosed by physicians were not different between the 2019 and 2020 groups (adjusted OR [aOR] = 1.05, 95% CI = 0.85–1.30, *p* = 0.647, Table 2). According to age and sex, the 19–39-year-old group demonstrated higher odds for depression diagnosed by physicians in the 2020 group than in the 2019 group (aOR = 1.58, 95% CI = 1.00–2.50, *p* = 0.049). All other age and sex subgroups did not show an association of depression diagnosed by physicians with depression in the 2020 group. The presence of current depression was not higher in the 2020 group than in the 2019 group. All age and sex subgroups did not show a relationship of current depression with depression in the 2020 group.

Stress at low, high, and very severe levels was not higher in the 2020 group when compared to the 2019 group (Table 3).

The values for planning suicide and attempting suicide were not high in the 2020 group compared to the 2019 group (Table 4).

## 4. Discussion

During the COVID-19 pandemic, the rates of depression, stress, and suicide attempts in Korean adults were not increased compared to the pre-pandemic era. Only the young adult population demonstrated a higher rate of depression during the COVID-19 pandemic than before the pandemic era. The present study improved previous findings on the impact of the COVID-19 pandemic on affective disorders by using a nationwide, representative population and adjusting numerous variables that could influence depression or stress levels.

A number of recent studies have reported the impacts of the COVID-19 pandemic on depression or stress [8,17,18,19,20]. A meta-analysis estimated that the prevalence of depression was 21.7% (95% CI = 18.3–25.2) in healthcare workers during the COVID-19 pandemic [17]. A cross-sectional survey indicated that more than 41.3% of participants suffered from depression, while less than 30% of participants reported stress [18]. In global review data, major depressive disorder increased during the COVID-19 pandemic, which was related to daily SARS-CoV-2 infection rates and immobility [19]. On the other hand, some regional reports described a nonsignificant increase in depression during the COVID-19 pandemic [21]. In an online survey in Cameroon, fear and depressive symptoms were high in participants with a history of quarantine, flu-like symptoms, and fear of COVID-19, while adequate COVID-19 information reduced the odds for depression [21]. In line with these results, another online survey predicted that the participants with viral symptoms and pre-existing mental health problems had a greater risk of depression [22]. On the other hand, the participants with old age, stable economic status, and adequate information about the pandemic had a lower risk of depression [22]. Therefore, it can be presumed that the impact of the COVID-19 pandemic on depression may depend on personal characteristics.

Contrary to the results from prior studies, the prevalence of depression,, stress, and suicide attempts were not higher during the COVID-19 pandemic for the current study. The honeymoon effects during the early pandemic period could have alleviated the risk of depression, stress, and suicidal attempts [23]. The public campaign for quarantine and health-related issues could strengthen the sense of belonging to the nation. To support this, a UK survey in pregnant women with COVID-19 reported that awareness of COVID-19 and related health information decreased depression at the end of the lockdown period, although the initial period of the COVID-19 lockdown could increase anxiety and depression, mainly due to the fear of infection and uncertainty [8]. In addition, the increased time spent with family may enhance family bonds and attenuate the stress from social activities. The lockdown of workplaces and schools due to the COVID-19 pandemic resulted in reduced burdens from jobs or school and increased time for leisure.

In the present study, a young adult population demonstrated an increased likelihood of being diagnosed with depression during the COVID-19 pandemic. Individual characteristics could influence the impact of the COVID-19 pandemic on depression. More active or extroverted individuals were reported to be more influenced by the COVID-19 lockdown. Prior researchers described the additional risks to mental health in the older population during the COVID-19 pandemic [24]. They insisted that excess morbidities and mortalities and the restrictions on medical access and availability in the older adult population have imposed additional risks to mental health during the COVID-19 pandemic [24]. However, although the elderly population is known to be vulnerable to depression, they have more experience coping with crisis and adversity than the younger population [25]. On the other hand, the young adult population has less coping capability than the old population. In addition, the COVID-19 pandemic induced the lockdown of the workplace and decreased the opportunity for job hunting, which can mediate depression among the young population.

This study used a nationwide population to compare cohorts before and during the COVID-19 pandemic. Socioeconomic status, such as income, employment, education level, house type, and marriage status, was examined and adjusted to minimize the potential confounding effects on depression and stress. In addition, a number of lifestyle factors, including obesity, sleep duration, smoking, and alcohol consumption, were considered. Because the cohort data were regularly validated and verified by a national statistician, risk selection bias was minimal. However, due to the cross-sectional study design, the causality of the COVID-19 pandemic for depression, stress, and suicide attempts could not be determined in the present study. The 2019 and 2020 cohort populations were independently selected, and a follow-up could not be conducted. In addition, the 2020 cohort represented the early COVID-19 pandemic period, and longer follow-up data may be needed to evaluate the long-term effects of the COVID-19 pandemic on depression, stress, and suicidal attempts. Lastly, this study was based on a Korean adult population; therefore, ethnic and regional differences in COVID-19 pandemic status should be considered when interpreting the present results [26].

## 5. Conclusions

Depression, stress, and suicidal attempts were not changed in Korean adults during the COVID-19 pandemic compared to before the COVID-19 pandemic. In the young adult population, the likelihood of being diagnosed with depression was higher during the COVID-19 pandemic than during the pre-pandemic period. Mental health care for susceptible populations should be considered during the COVID-19 pandemic.

## Figures and Tables

**Table 1:** General characteristics of participants.

Characteristics		Year		*p* Value *
		2019	2020	
Age (yrs, mean, SD)		51.7 (16.9)	52.0 (17.3)	0.799
Age groups (yrs, n, %)				0.892
	19–39	1656 (34.9)	1552 (34.3)	
	40–59	2258 (39.3)	2009 (39.2)	
	≥60	2213 (25.8)	2185 (26.5)	
Sex (n, %)				0.695
	Males	2719 (49.7)	2590 (50.0)	
	Females	3408 (50.3)	3156 (50.0)	
Income (mean, SD)		3.2 (1.4)	3.3 (1.4)	0.551
Income group (n, %)				0.938
	1 (lowest)	932 (11.4)	804 (11.0)	
	2	1131 (16.5)	1043 (15.6)	
	3	1240 (21.5)	1183 (21.3)	
	4	1383 (24.6)	1332 (25.0)	
	5 (highest)	1441 (26.0)	1384 (27.1)	
Employment (n, %)				0.068
	Unemployed	2557 (1.2)	2520 (1.3)	
	Employed	3570 (1.9)	3226 (1.8)	
Educational status (n, %)				0.498
	Elementary school or under, Unknown	1331 (16.2)	1297 (16.9)	
	Junior high school	554 (7.5)	517 (7.1)	
	High school	1963 (34.1)	1884 (36.2)	
	College or over	2279 (42.1)	2048 (39.7)	
House type (n, %)				0.678
	Detached house	1906 (27.7)	1859 (27.7)	
	Condominium	3343 (57.0)	3203 (60.2)	
	Raw houses	829 (14.5)	655 (11.7)	
	Others	49 (0.8)	29 (0.4)	
Marriage status (n, %)				0.103
	Married	4210 (66.6)	3744 (63.5)	
	Unmarried	861 (10.5)	843 (10.9)	
	Unknown	1056 (22.9)	1159 (25.6)	
Body mass index (mean, SD)		23.9 (3.6)	24.2 (3.7)	0.001 *
Smoking status (n, %)				
	Nonsmoker	3635 (56.5)	3451 (56.8)	
	Past smoker	1418 (23.1)	1331 (23.8)	
	Current smoker	1074 (20.3)	964 (19.4)	
Alcohol consumption (n, %)				0.070
	Nonconsumer	2824 (42.1)	2805 (44.4)	
	1 to 5 times/mo	2005 (36.0)	1777 (33.3)	
	≥2 times/week	1298 (21.9)	1164 (22.3)	
Sleep duration (mean, SD)		6.9 (1.4)	7.0 (1.4)	0.097
Hypertension (n, %)		1549 (20.4)	1481 (20.7)	0.817
Dyslipidemia (n, %)		1190 (16.4)	1280 (18.5)	0.021 *
Stroke (n, %)		138 (1.8)	119 (1.5)	0.273
Ischemic heart disease (n, %)		181 (2.4)	175 (2.1)	0.390
Diabetes mellitus (n, %)		600 (8.1)	645 (9.1)	0.137
Chronic kidney disease (n, %)		24 (0.4)	102 (1.5)	<0.001 *
Depression (n, %)				
	Doctor-diagnosed	285 (4.1)	292 (4.5)	0.326
	Current	159 (2.2)	182 (2.7)	0.115
Stress (n, %)				0.741
	None	965 (13.9)	901 (13.4)	
	A little	3533 (57.4)	3270 (57.7)	
	A lot	1347 (23.8)	1295 (23.6)	
	Very severe	282 (4.8)	280 (5.3)	
Suicide (n, %)				
	Planning suicide	83 (1.3)	109 (1.7)	0.126
	Attempting suicide	25 (0.4)	26 (0.4)	0.830

SD: standard deviation. * The general characteristics in 2019 and 2020 were compared using linear regression analysis with complex sampling for continuous variables and the chi-square test with Rao–Scott correction for categorical variables. Significance at *p* < 0.05.

**Table 2:** Odds ratios (95% confidence intervals) for depression (doctor-diagnosed depression, current depression) in 2020 compared to 2019 with subgroup analyses according to age and sex.

Characteristics			Odds Ratios for Depression in 2020 Compared to 2019			
			Crude	*p* Value *	Adjusted †	*p* Value *
Doctor-diagnosed depression						
	Total participants (n = 11,873)		1.11 (0.90–1.37)	0.328	1.05 (0.85–1.30)	0.647
	Age					
		19–39 years old (n = 3208)	1.81 (1.17–2.81)	0.008 *	1.58 (1.00–2.50)	0.049 *
		40–59 years old (n = 4267)	0.84 (0.59–1.19)	0.331	0.78 (0.55–1.09)	0.148
		≥60 years old (n = 4398)	0.94 (0.68–1.30)	0.696	0.92 (0.66–1.29)	0.633
	Sex					
		Males (n = 5309)	1.11 (0.76–1.62)	0.586	1.00 (0.69–1.46)	0.989
		Females (n = 6564)	1.12 (0.87–1.42)	0.379	1.06 (0.83–1.36)	0.625
Current depression						
	Total participants (n = 11,873)		1.24 (0.95–1.63)	0.117	1.19 (0.91–1.56)	0.213
	Age					
		19–39 years old (n = 3208)	2.01 (1.15–3.52)	0.014	1.75 (0.97–3.18)	0.064
		40–59 years old (n = 4267)	0.90 (0.59–1.39)	0.642	0.84 (0.54–1.30)	0.429
		≥60 years old (n = 4398)	1.06 (0.69–1.64)	0.777	1.08 (0.69–1.71)	0.736
	Sex					
		Males (n = 5309)	1.19 (0.75–1.90)	0.462	1.06 (0.66–1.69)	0.817
		Females (n = 6564)	1.27 (0.93–1.74)	0.130	1.25 (0.90–1.72)	0.183

Abbreviation: BMI, body mass index. * Logistic regression, significance at *p* < 0.05; † adjusted for age, sex, income, employment, educational status, house type, marriage status, BMI, smoking status, alcohol consumption, sleep duration, hypertension, dyslipidemia, stroke, ischemic heart disease, diabetes mellitus, and chronic kidney disease.

**Table 3:** Odds ratios (95% confidence intervals) for stress in 2020 compared to 2019.

Characteristics		Odds Ratios for Stress Level in 2020 Compared to 2019			
		Crude	*p* Value *	Adjusted †	*p* Value *
Total participants (n = 11,873)					
	A little stress	1.04 (0.90–1.21)	0.587	1.08 (0.94–1.24)	0.300
	A lot of stress	1.03 (0.85–1.23)	0.792	1.07 (0.90–1.27)	0.458
	Very severe stress	1.15 (0.89–1.49)	0.283	1.19 (0.93–1.54)	0.168

Abbreviation: BMI, body mass index. * Multinomial logistic regression, significance at *p* < 0.05; † adjusted for age, sex, income, employment, educational status, house type, marriage status, BMI, smoking status, alcohol consumption, sleep duration, hypertension, dyslipidemia, stroke, ischemic heart disease, diabetes mellitus, and chronic kidney disease.

**Table 4:** Odds ratios (95% confidence intervals) for suicide (planning suicide, attempting suicide) in 2020 compared to 2019.

Characteristics		Odds Ratios for Suicide in 2020 Compared to 2019			
		Crude	*p* Value *	Adjusted †	*p* Value *
Total participants (n = 11,873)					
	Planning suicide	1.34 (0.92–1.96)	0.130	1.31 (0.90–1.90)	0.161
	Attempting suicide	0.93 (0.50–1.76)	0.831	0.78 (0.41–1.48)	0.438

Abbreviation: BMI, body mass index. * Logistic regression, significance at *p* < 0.05; † adjusted for age, sex, income, employment, educational status, house type, marriage status, BMI, smoking status, alcohol consumption, sleep duration, hypertension, dyslipidemia, stroke, ischemic heart disease, diabetes mellitus, and chronic kidney disease.

## Data Availability

Restrictions apply to the availability of these data. Data were obtained from the Korea National Health and Nutrition Examination Survey and are available at https://knhanes.kdca.go.kr/knhanes/main.do (accessed on 20 January 2022).
